# Study on the Microstructure and Mechanical Properties of Diamond Particle-Reinforced Copper-Iron Sandwich Composites Prepared by Powder Metallurgy

**DOI:** 10.3390/ma15072424

**Published:** 2022-03-25

**Authors:** Jian Sun, Boyi Jiang, Wanzhong Li, Xiaole Cheng, Hui Liu, Ziyang Li

**Affiliations:** 1 School of Mechanical and Electrical Engineering, Xi’an Polytechnic University, Xi’an 710048, China; jby199711@163.com (B.J.); chengxiaole@xpu.edu.cn (X.C.); huiliu@xpu.edu.cn (H.L.); lzyli0501@163.com (Z.L.); 2 Xi’an Key Laboratory of Modern Intelligent Textile Equipment, Xi’an 710048, China; 3 School of Mechanical Engineering, Xi’an Shiyou University, Xi’an 710065, China

**Keywords:** synthetic diamond, copper-iron, microstructure, powder metallurgy

## Abstract

Synthetic diamond particle-reinforced copper-iron composites (SD/Cu-Fe) were produced by the powder metallurgical method for stone cutting applications, and the microstructure, density, compactness, hardness, flexure strength, and wear resistance of the composites were characterized in this work. The results showed that the diamond particles were relatively uniformly distributed in most areas of the copper matrix and the crystal shape of diamond particles were relatively intact in the sintering temperature range from 740 °C to 780 °C. The interfaces between the diamond particles and copper matrix, as well as the interfaces between the copper matrix and iron layer, were well bonded without significant gaps. The physical properties of composites increased first and then decreased with the sintering temperature. When the sintering temperature was 770 °C, the related properties reached the best. Diamond played a key role in improving the properties of the SD/Cu-Fe sandwich composite. This work provides a basis for the research and development of high-performance diamond-reinforced copper-based iron sandwich composites.

## 1. Introduction

The diamond-reinforced composite is a kind of high-performance composite with rapid development and a wide range of applications. It has the advantages of high strength, good heat resistance, sharp cutting, and high grinding efficiency, and it is not easy to plug. However, due to the greater brittleness and poor impact resistance of conventional diamond-reinforced composites, its application in the fields of cutting tools and abrasives has great limitations [1]. Diamond has a high thermal conductivity (600~2000 W/(m·K)) and low thermal expansion coefficient (about 1 × 10^−6^ K^−^^1^) [2], while copper has a high thermal conductivity (400 W/(m·K)), high thermal expansion coefficient (about 1.7 × 10^−5^ K^−^^1^), low price, and easy processing characteristics. The diamond particle-reinforced copper matrix composite prepared by combining the two characteristics has the advantages of a high thermal conductivity and adjustable thermal expansion coefficient, and it is widely used in many industrial fields such as electronic packaging [3], instruments, and meters.

With the development and application of technology, the composite of diamond and copper materials also brings a series of problems worth studying, such as the development of a more appropriate preparation process, and designing a better material formulation and structure, so that copper and diamond can be well combined and the composite has better comprehensive properties, especially thermal properties and mechanical characteristics. Therefore, the research and application of diamond particle-reinforced copper matrix composites have always been the key research fields of the majority of researchers in electronic packaging and cutting tools industries. As early as 1995, researchers in relevant technical fields successfully developed diamond-reinforced copper matrix composites called Dymally. The thermal conductivity of the composite material can reach 420 W/(m·K), and the average thermal expansion coefficient of the material fully meets the performance requirements of relevant components when the temperature does not exceed 200 °C. However, the preparation process of the material is relatively complex and the production cost is high [4,5]. With the continuous development and improvement of the preparation technology of metal matrix composites, the interfacial bonding strength between diamond and the copper matrix seriously affects the comprehensive properties of copper matrix composites. Some scholars have optimized the bonding strength by adding alloying elements. Schubert [6], Zeinab [7], Abyzov [8], and others successively added Cr to the copper matrix or plated metal or alloy elements such as NiWB on the diamond surface to form metal compounds between diamond and the copper matrix during high-temperature preparation to improve the bonding strength between the copper matrix and diamond particles. In order to improve the bonding strength and reduce the performance degradation caused by element diffusion and the stress caused by the thermal mismatch of materials, scholars have carried out a lot of research on composite structures. Silva [9] proposed a Ni/Cu/Ti multilayer system to solve the problem of the lack of adhesion and growth rates. In his study, he mentioned that adding a thick soft copper layer in the structure can better relieve the problem of the thermally induced stresses during the cooling stage of the MPCVD (Microwave Plasma Chemical Vapor Deposition) process. Then, Silva [10] added a Ni/Cu layer with embedded diamond particles between the diamond films and steel substrates. With this interlayer, a range of diamond film growth parameters were found to give crack-free and nearly strain-free diamond continuous coatings. Therefore, adding a flexible layer is a powerful method to solve the problems of bonding strength, thermal mismatch stress, and diffusion. This provides an idea for the design of high-performance composite materials. Some scholars have studied the preparation process and parameters to improve the comprehensive properties of composites. Hu et al. [11] prepared diamond particle-reinforced copper matrix composites with different particle sizes by the high-pressure method, which provided a new idea for the preparation of new copper matrix composites, and the reduction in steel particle size could enhance the hardness of the composites. Wu [12] prepared Al/diamond composites by the pressure infiltration method. It was found that the mechanical properties of the composites gradually increased with the decrease in diamond particle size. Zhu [13,14] prepared chromium-coated diamond particles-reinforced copper matrix composite heat dissipation materials with excellent thermal physical properties by spark plasma sintering, which realized full chromium coating on the surface of diamond particles and good interface bonding with copper matrix metal materials. A new molten salt method was proposed to prepare surface-modified diamond particles by in situ reaction. The surface-modified diamond particles-reinforced metal matrix composites had high thermal conductivity. Zhao [15] prepared diamond/copper composites with different particle sizes and volume fractions by powder metallurgy. The optimized process scheme of vacuum secondary hot pressing was proposed to improve the preparation process of diamond/copper composites. Zhang [16] and others successfully prepared diamond/copper matrix composites with high thermal conductivity by spark plasma sintering, realized the preparation of copper matrix composites with an ultra-high content of reinforcements, and effectively improved the compactness of diamond/copper matrix composites. Long [17] et al. prepared diamond/copper matrix composites by spark plasma sintering, and they found that when the mass fraction of diamond was 1%, the maximum tensile strength of diamond/copper matrix composites was 221.35 MPa. When the mass fraction was 1.5%, the optimum density was 95.52%. Chen [18] et al. prepared diamond/copper composites with bimodal particle size by pressure infiltration, and the influence of diamond particles with different volume ratios of 40 μm and 100 μm on the properties of copper matrix composites was investigated. Wang [19] explored the relationship between the interface structure and characteristic parameters of composites by two different interface modification methods: copper matrix alloying and diamond surface metallization. Obviously, most studies were still focused on improving the interfacial bonding strength and obtaining better thermodynamic properties of diamond/copper matrix composites. There are a lot of research areas for the mechanical properties and wear resistance of diamond/copper matrix composites in tool applications.

In this work, synthetic diamond-reinforced copper matrix/iron sandwich structure composites (SD/Cu Fe sandwich composites), which can be used for stone cutting, were prepared by powder metallurgy using artificial diamond particles with large particle size (the particle size was between 200 μm and 250 μm) and copper powder, zinc powder, and iron powder. The microstructure, bending resistance, and wear resistance were studied. It provides a research basis for analyzing and developing high-performance diamond-reinforced composite tools.

## 2. Sample Preparation and Testing

### 2.1. Sample Preparation

The method of powder metallurgy was used to prepare SD/Cu-Fe composites. The preparation process mainly included ball milling, powder mixing, blank pressing, and sintering. (1) The required raw materials were prepared by the powder mixing process for the test. The raw materials included copper powder with a particle size of 160 mesh, zinc powder and iron powder with a particle size of 200 mesh, and synthetic diamond with a particle size of 200–250 μm. According to the mass percentage of each component: Zn: 18–22%; Fe: 36–38%; diamond: 8–12%; the rest: copper and inevitable impurities. The presence of impurities may cause defects in the bonding interface of SD/Cu-Fe sandwich composites and decrease the mechanical properties, so it is necessary to reduce the presence of impurities as much as possible. After weighing, it was poured into the ball mill jar, and certain amounts of 4 mm, 6 mm, and 8 mm Agate balls were added, which were doped at a ratio of 3:5. In order to prevent oxidation, the powder was made in an argon atmosphere, the ball milling time was set to 6 h, the ball milling speed was set to 300 r/min, the forward and reverse mixing was used, and the interval time was 30 s. After the AD/Cu composite powder was successfully prepared, it was necessary to put the composite powder into a vacuum bag and seal it to prevent the composite powder from being oxidized or introducing other impurities during storage. (2) Then, the copper matrix composite powder and iron powder were compacted in a 15 mm × 22 mm graphite mold. First, a layer of copper matrix composites powder with a thickness of 2.8–3 mm was laid in the mold, the initial pressure was carried out under a pressure of 200 MPa, and the pressure holding time was 3 min. Then, a layer of iron powder initial pressure was also carried out under a pressure of 200 MPa for 3 min. The SD/Cu-Fe sandwich composites were made by repeating the above steps. (3) Sintering was completed. The formed sample was put into the tubular furnace and sintered in hydrogen atmosphere. The initial temperature of the tubular furnace was 200 °C and the heating rate was 15 °C/min. The temperature rose to 500 °C and lasted for 2 h. After that, the compact was repressed under the pressure of 750 MPa for 3 min. It was kept at 740–780 °C for 2 h for secondary sintering after that. Then, it was cooled down to less than 100 °C with the furnace and air-cooled to room temperature. After sintering, the required test samples could be obtained. There were three layers of iron interlayers in the middle of four layers of diamond-reinforced copper matrix composites.

### 2.2. Testing Method

X-ray diffraction analysis, referred to as XRD [20], is an analytical method to measure the internal atomic structure of the object to be measured by X-ray, which can be used for qualitative and quantitative analysis of the material. The DX-2700BH X-ray diffractometer produced by Dandong Haoyuan Instrument Co., Ltd. (Dandong, China) was used to analyze and characterize the phase of composites. The test parameters were as follows: the tube voltage was 30 kV, the tube current was 20 mA, the starting angle of scanning was 30°, the ending angle was 90°, the measurement time was 0.3 s, and the step width angle was 0.03°.

A scanning electron microscope, also known as SEM [21], was used to analyze and characterize the micromorphological characteristics of the surface to be tested. In this work, the imported FlexSEM1000 compact intelligent scanning electron microscope (Japan Hitachi Nake high-tech enterprise, Ibaraki Prefecture, Japan) was used to test and characterize the microstructure of SD/Cu-Fe sandwich composites. The test voltage was 20 kV, and the electron gun was pre-aligned with the tungsten filament. The composition of SD/Cu-Fe sandwich composites was analyzed by an energy spectrometer equipped with a QUANTA-450-FEG field emission scanning electron microscope (Switzerland Textest Company, Schwerzenbach, Switzerland). The distribution of each component of the SD/Cu-Fe sandwich composite was more intuitively characterized by surface scanning. The working voltage and current of the field emission scanning electron microscope were 20 kV and 80 μA, respectively. The signal receiving time was 300 s.

The density of AD/Cu-Fe interlayer composites was measured and calculated by using the principle of the Archimedes drainage method combined with the hydrostatic weighing method. The specific measurement and calculation process can be divided into four steps: (1) Ultrasonic cleaning: The AD/Cu-Fe interlayer composite samples were ultrasonically cleaned with a KQ-700VDB dual-frequency numerically controlled ultrasonic cleaner (Kunshan Ultrasonic Instruments Co., Ltd., Kunshan, China), and the time was set to 10 min. Its purpose was to remove impurities on the surface of the sample and residual impurities in the cavity, thereby reducing the calculation error of density. (2) Exhaust gas: The cleaned AD/Cu-Fe interlayer composite sample was put into boiled deionized water and let to stand for 5~10 min to discharge the residual gas inside the sample. (3) Sample weighing: The size of the AD/Cu-Fe sandwich composite material sample was 22 mm × 12 mm × 9 mm. According to the size of the sample and the weight of the sample, the balance and other auxiliary tools with suitable range and accuracy were selected. The sample was tied to the spreader and put into water for weighing. After the sample was removed, the mass of the spreader was weighed again. Then, the AD/Cu-Fe sandwich composite sample was taken out from the deionized water and dried, placed on the same balance, and weighed. (4) The sample density was calculated by using the principle of the Archimedes drainage method.

The PRHR-150A Rockwell hardness tester (Shandong iPRE Detection Technology Co., Ltd, Weifang, China) was selected to measure the macrohardness of SD/Cu-Fe sandwich composites. The hardness measurement error was 0.5 HRC. During the test, the initial test force (preload) was 10 kg, the loading force was 150 kg, and the pressurization time was 8 s. In order to ensure the accuracy of the measurement results, 10 measurement points were selected at equal intervals from left to right and from top to bottom for each sample, and the extreme points (a maximum and a minimum) were removed after the measurement. Finally, the average value of the remaining groups of data was calculated as the macrohardness value of the final SD/Cu-Fe sandwich composites.

**Figure 1:**
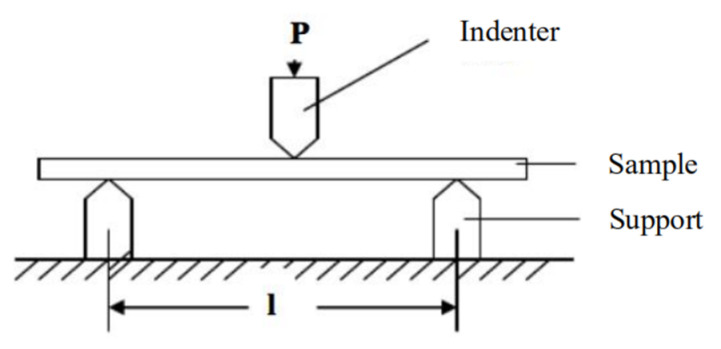
Schematic diagram of bending strength test.

The bending strength (also known as the flexural strength) is one of the very important indexes in the mechanical properties of cutting tools materials. The three-point bending loading method is usually used to test the bending strength of cutting tools materials. The test principle of the three-point bending loading method was as follows: make the sample to be tested into a strip block shape of 22 mm × 12 mm × 9 mm; then, clamp it on the support, apply the load from top to bottom with the indenter after fixing, and stop the test after the sample is completely broken. The test schematic is shown in Figure 1.

A WDW-3050 microcomputer-controlled electronic universal testing machine (Testing Instrument Research Institute of Changchunkexin Company, Changchun, China) was selected. The surface of the sample to be tested was polished to eliminate the influence of surface microcracks on the test results. When clamping the sample, the span between the two fulcrums of the support was adjusted according to the specific size of the sample, the span dimension L was 18 mm, the fixing screws of the support were tightened, and the sample was clamped in the fixture of the support. After the experiment started, the downward movement speed of the indenter was set to 5 mm/min.

The ML-100 abrasive wear machine (Jinan Hansen Precision Instrument Co., Ltd, Jinan, China) was selected for the testing of wear resistance of AD/Cu-Fe sandwich composites through the pin-on-disk wear test. After the experiment started, each sample moved from the center position of the disc to the edge position, and the whole running-in stroke was one grinding course of the sample. After measurement, the inner diameter of the formed annular domain was 31 mm, and the outer diameter was 229 mm. The feed rate selected in this experiment was 2 mm/rot and the disc speed was 60 rot/min. The grinding path in the test was 18.38 m.

## 3. Microstructure Properties

### 3.1. Composition Analysis of AD/Cu-Fe Sandwich Composites

**Figure 2:**
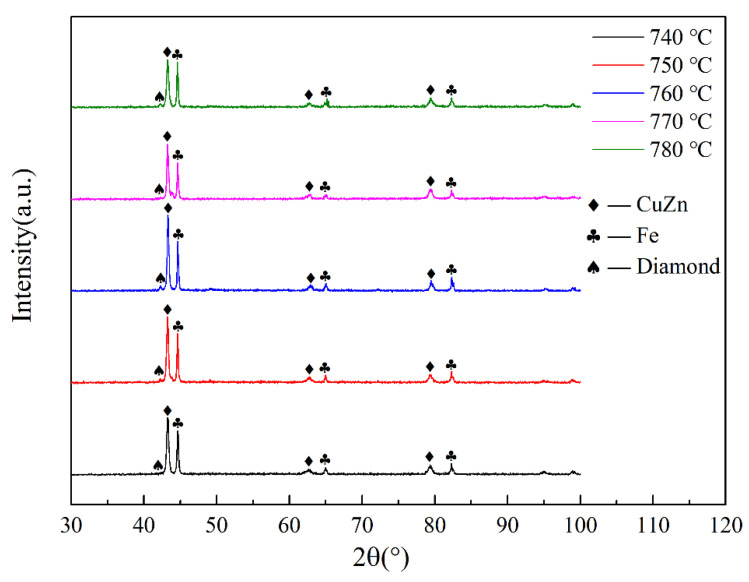
XRD pattern of AD/Cu-Fe sandwich composite.

The XRD patterns of AD/Cu-Fe sandwich composites are shown in Figure 2.

Compared with the standard PDF (Powder Diffraction File) card, the results showed that the diffraction peaks contained three phases of CuZn, Fe, and diamond. The diffraction peaks at 2θ values of 43.3°, 63°, and 79.5° correspond to the (110), (200), and (211) crystal planes of CuZn, and the diffraction peaks at 2θ values of 44.8°, 65°, and 82.4° correspond to the (110), (200), and (211) crystal planes of Fe. The diffraction peak intensity of diamond was weaker than those of CuZn and Fe, the 2θ value was about 42°, and there was a characteristic diffraction peak, corresponding to the (011) crystal of diamond. There were characteristic peaks in the 2θ value between 43.5° and 44.5°, corresponding to the (012) crystal plane of diamond.

### 3.2. Elemental Analysis of AD/Cu-Fe Sandwich Composites

It was found that the AD/Cu-Fe interlayer composite mainly contained four elements: C, Cu, Zn, and Fe. The distribution was analyzed under five temperature gradients from 740 °C to 780 °C. The analysis results showed that the C diffused to a certain extent in the AD/Cu-Fe interlayer composite material in this temperature range, but there was no obvious diffusion area at the junction of the diamond particles and the matrix material. There was no obvious diffusion of Cu in AD/Cu-Fe interlayer composites. This was because the solubility of copper in iron was extremely low, and the combination of the copper-iron alloy was not like other alloys, it was a special form of existence [22]. There was no diffusion phenomenon of Zn in the Fe interlayer, but it was evenly distributed in the Cu matrix. The Fe diffused obviously in the copper matrix, and the α-Fe phase in the Cu matrix presented a dendrite that was not fully developed [23].

### 3.3. Distribution of Diamond Particles in Copper Matrix

**Figure 3:**
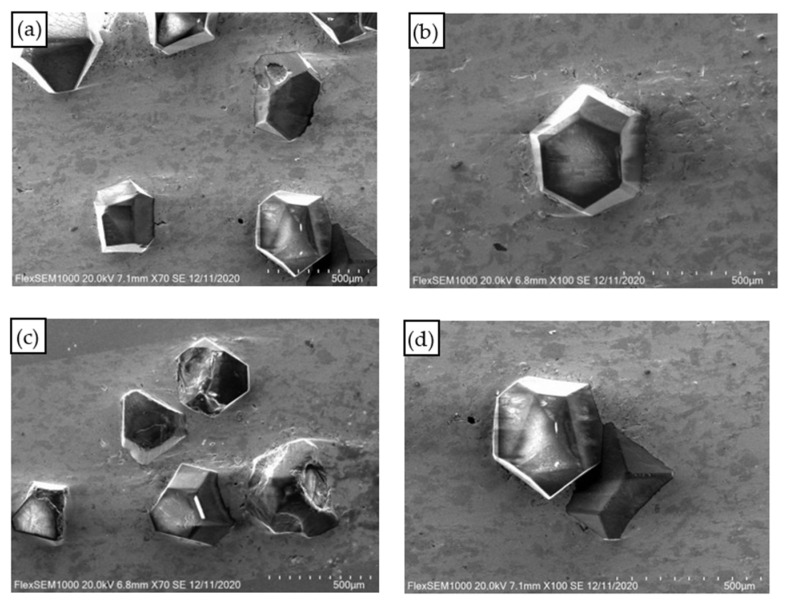
The distribution of diamond particles in the copper matrix. (**a**) High volume distribution; (**b**) Low volume distribution; (**c**) Fragmentation of diamond particles; (**d**) Diamond particle buildup.

When the sintering temperatures were 740 °C, 750 °C, 760 °C, 770 °C, and 780 °C, the distribution of diamond particles was relatively uniform and the volume fraction was relatively high in most areas of the copper matrix, as shown in Figure 3a. The volume fraction of diamond particles in some regions was low, as shown in Figure 3b.

Most of the diamond particles were relatively complete in crystal form, and only a small amount of diamond particles appeared fragmented and accompanied by the phenomenon of diamond particle stacking, as shown in Figure 3c,d. The uneven distribution of diamond particles in the copper matrix may be because: (1) the diamond particles were large and the volume fraction was high; (2) the balls were relatively small or the ball milling time was short during ball milling.

### 3.4. Bonding Interface between Diamond Particles and Copper Matrix

**Figure 4:**
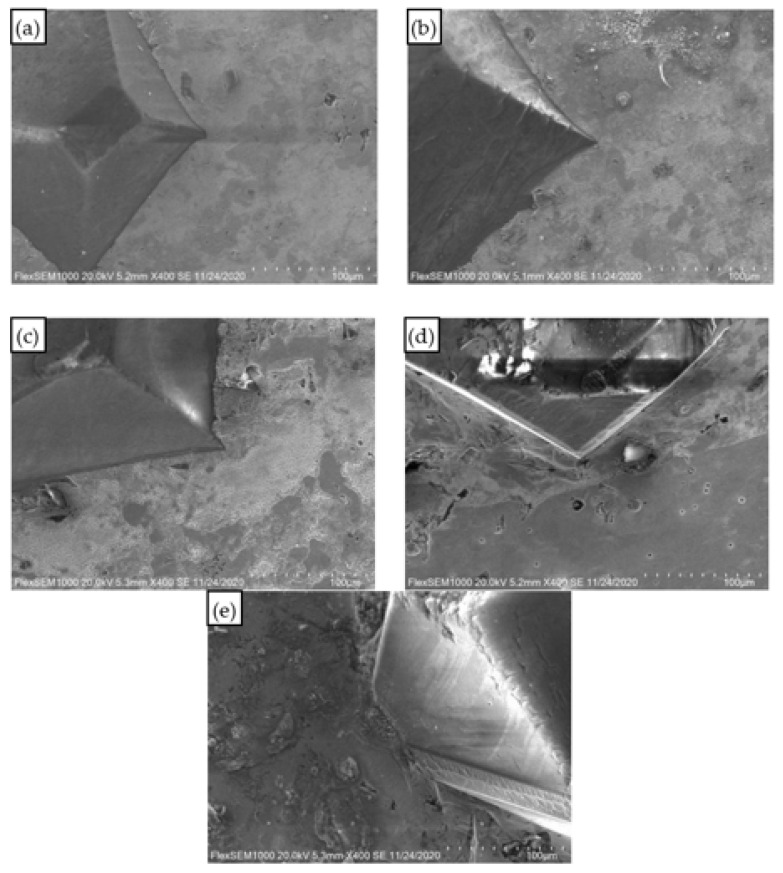
Bonding interface between diamond particles and copper matrix. (**a**) 740 °C; (**b**) 750 °C; (**c**) 760 °C; (**d**) 770 °C; (**e**) 780 °C.

At five sintering temperatures of 740 °C, 750 °C, 760 °C, 770 °C, and 780 °C, the interface between the diamond and copper matrix was well bonded without an obvious gap, as shown in Figure 4. A good bonding interface will reduce the thermal resistance of the composite interface, improve the thermal conductivity of the composite, and improve the density of the composite, which is conducive to the improvement of cutting tools performance.

### 3.5. Bonding Interface between Copper Matrix and Iron Layer

**Figure 5:**
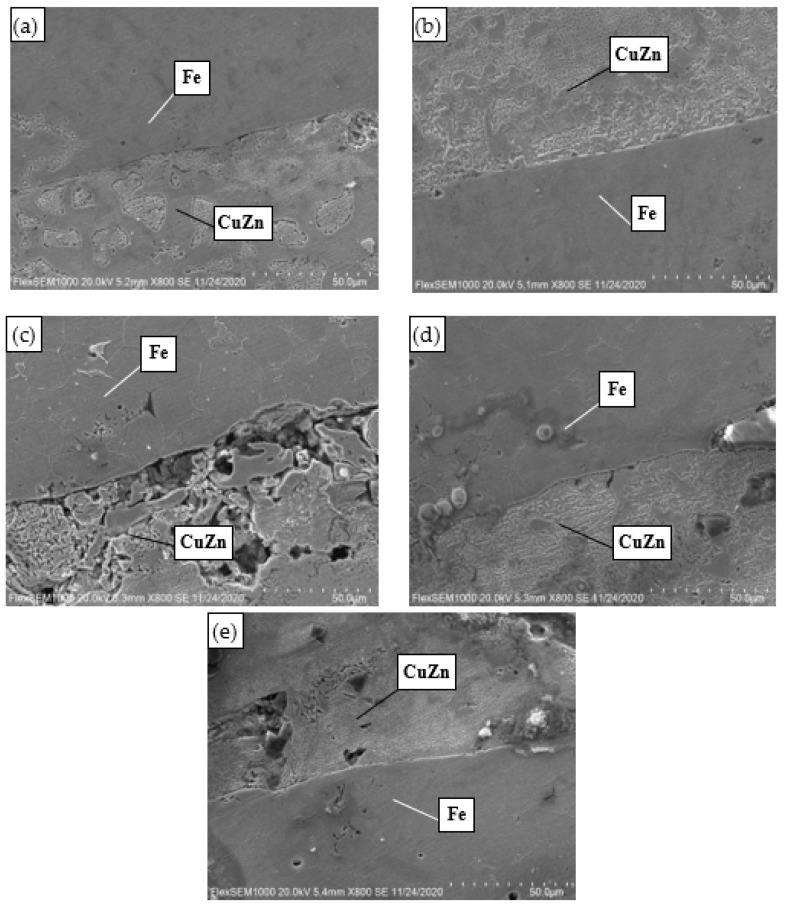
Bonding interface between copper matrix and iron interlayer: (**a**) 740 °C; (**b**) 750 °C; (**c**) 760 °C; (**d**) 770 °C; (**e**) 780 °C.

The interface morphology of the copper matrix and iron layer under five sintering temperatures is shown in Figure 5. It can be seen from Figure 5a,b that when the sintering temperatures were 740 °C and 750 °C, the interfaces between the copper matrix and iron layer were well bonded, there were few pores between the copper matrix and iron layer, and the compactness of the composite was good. When the sintering temperature was 760 °C, as shown in Figure 5c, the bonding performance at the interface between the copper matrix and iron layer was relatively poor, large pores appeared in some areas, and the density of the copper matrix was poor. The reason for this phenomenon may be that the powder was not paved during the pressing process, resulting in defects in the blank. When the sintering temperatures were 770 °C and 780 °C, as shown in Figure 5d,e, the interfaces between the copper matrix and iron layer were well bonded.

## 4. Macroscopic Properties

The key characteristic parameters of materials used in cutting tools were investigated, mainly including density, densification, hardness, bending strength, and wear resistance.

### 4.1. Density and Compactness

**Figure 6:**
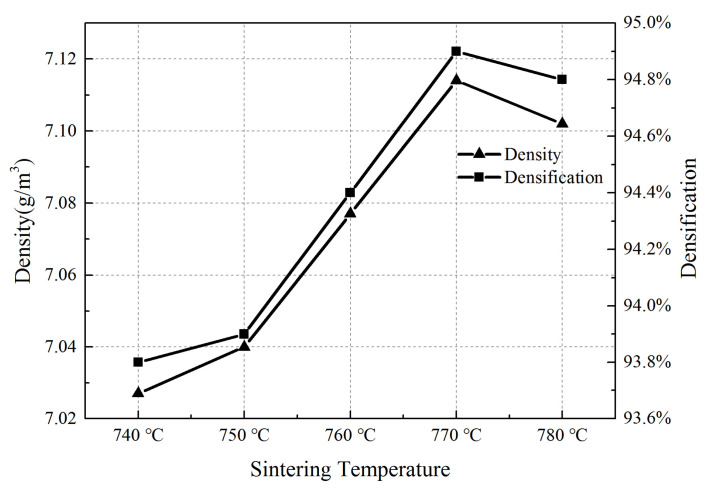
Density and compactness of AD/Cu-Fe sandwich composite material.

The density and densification of AD/Cu-Fe sandwich composites are shown in Figure 6. It can be seen from the figure that the density and densification of AD/Cu-Fe interlayer composites sintered at 740 °C were smallest, which were 7.0727 g/cm^3^ and 93.8%, respectively. When the sintering temperature increased to 770 °C, the density and densification of AD/Cu-Fe interlayer composites reached the maximum value, which were 7.114 g/cm^3^ and 94.9%, respectively. The density and densification of SD/Cu-Fe composites increased first and then decreased with temperature, a trend consistent with the results in [15]. The main reason is that with the gradual increase in temperature, the interior of the SD/Cu-Fe composite becomes denser, and the interface condition is optimized. When the sintering temperature exceeds 770 °C, the over-sintering phenomenon will cause defects at the interface and reduce the densification.

### 4.2. Hardness

Hardness is the basis of characterizing the deformation resistance of a material surface [24], and the stronger the deformation resistance of the surface, the higher the hardness. The addition of diamond can improve the hardness of the composites, and because the Vickers hardness is relatively common in laboratory analysis, Rockwell hardness was converted into Vickers hardness in this work. The hardness of SD/Cu-Fe composites at different sintering temperatures of 740–780 °C is shown in Table 1.

The hardness of the SD/Cu-Fe sandwich composites also increased first and then decreased with temperature. The hardness value was as high as 700 HV at 780 °C and lowest at 740 °C, which was 640 HV. The highest hardness was 940 HV at 770 °C. The addition of diamond increased the number of interfaces between diamond and the copper matrix, which would weaken the movement of dislocation. On the other hand, due to the different coefficient of thermal expansion between diamond and copper, a higher stress concentration would occur in the material, and these stresses would be released during the cooling process after sintering, resulting in the generation of strain, which is called thermal mismatch strain. During relaxation, these strains will release dislocation loops. Diamond plays the role of fixing dislocations in the process of plastic deformation, to improve the hardness of the composites.

### 4.3. The Flexure Strength

**Figure 7:**
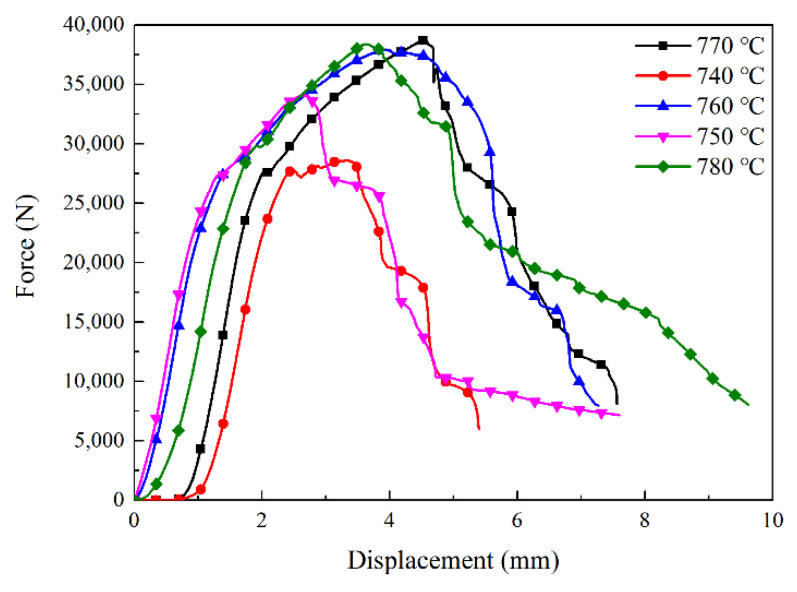
Diagram of the force–displacement relationship in the three-point bending load test of the SD/Cu-Fe sandwich composite.

After the measurement of the three-point bending load test, the force–displacement relationship of each group of samples in the SD/Cu-Fe sandwich composite is shown in Figure 7. The results are consistent with the trend in the literature [25]. It can be seen from the figure that the sample experienced six stages during the test: elastic deformation–plastic deformation, cracking–crack propagation–secondary elastic deformation–secondary plastic deformation, and cracking–crack propagation. The reason for the above-mentioned fracture phenomenon of the sample was that during the experiment, the stress in the loaded area of SD/Cu-Fe sandwich composite (i.e., the middle position of the sample) was concentrated, resulting in elastic deformation. As the stress increased to a certain extent, cracks occurred in the middle section of the lower surface of the SD/Cu-Fe sandwich composite. Then, the crack expanded upward until the sandwich composites near the lower surface broke for the first time. At this time, the diamond/Cu sandwich and iron layer on the upper surface continued to maintain elastic deformation and quickly change to the plastic deformation stage and produce cracks. With the continuous increase in stress, the final sample broke, and with the increase in the sintering temperature, the mechanical properties also increased. After the temperature reached 760 °C, the mechanical properties no longer increased significantly.

The flexure strength of each group of samples was calculated according to the calculation formula of the bending strength of rectangular section samples, and the results are shown in Table 2.

According to the calculation results of bending strength, the sintering temperature was in the range of 740–780 °C. With the increase in sintering temperature, the flexure strength of the SD/Cu Fe sandwich composite increased first and then decreased. When the sintering temperature was 770 °C, the bending strength reached the maximum value of 1076 MPa, the crystallinity of Fe and CuZn alloys was best, and the SD/Cu-Fe sandwich composites showed the best bending strength. When the sintering temperature continued to rise, there was too much Fe in the copper matrix, which inhibited the crystallization process of the CuZn phase. Therefore, the flexure strength of SD/Cu Fe sandwich composites decreased again.

### 4.4. Wear Resistance

**Figure 8:**
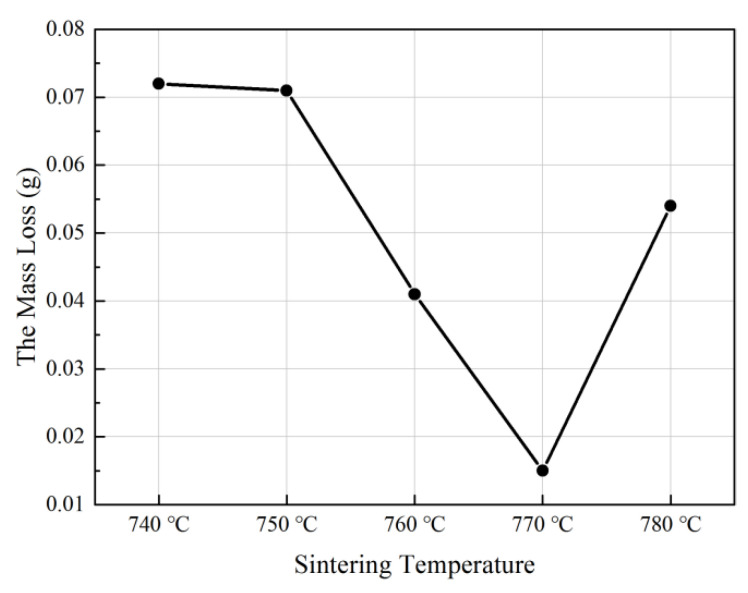
Wear resistance of SD/Cu-Fe sandwich composites.

The wear sample size was 22 mm × 15 mm × 9 mm. Figure 8 shows the variation trend of mass loss under the same wear conditions. It can be seen from the figure that in the temperature range of 740–780 °C, the mass loss of the SD/Cu-Fe sandwich composite decreased first and then increased with the sintering temperature. When the sintering temperature reached 770 °C, the minimum wear amount of the SD/Cu-Fe sandwich composite was 0.015 g. Compared with the changing trend of hardness, the variation trend of wear was opposite. When the temperature reached 770 °C, the hardness of SD/Cu-Fe sandwich composites reached the maximum. Therefore, when the hardness of composites increased, its wear resistance improved more. It can be concluded that the best sintering temperature of the SD/Cu-Fe sandwich composite in the five groups of sintering temperature was 770 °C.

**Figure 9:**
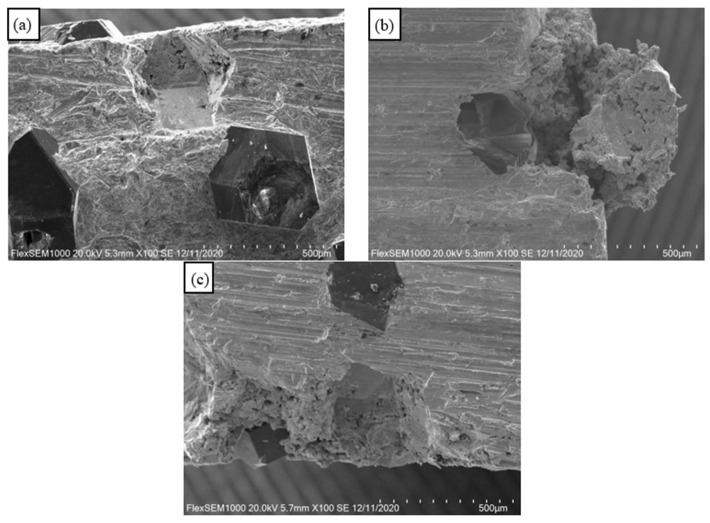
Defects of SD/Cu-Fe sandwich composite. (**a**) 740 °C; (**b**) 750 °C; (**c**) 780 °C.

By observing the wear morphology of SD/Cu-Fe sandwich composites, it was found that when the sintering temperatures were 740 °C and 780 °C, peeling pits of diamond particles appeared on the surface of the copper matrix at the edge of two groups of SD/Cu-Fe sandwich composites, as shown in Figure 9a,c. 

The results showed that for sintering at 740 °C and 780 °C, the interfacial bonding strength between diamond particles and the copper matrix was poor, and the holding force of the copper matrix on diamond particles was insufficient. As shown in Figure 9b, when the sintering temperature was 750 °C, the copper matrix material at the edge of the sandwich composite appeared spalling. Usually, there are two reasons for this phenomenon: the powder is not paved smoothly during the blank pressing process of the SD/Cu-Fe sandwich composite, and there is not enough composite powder at the edge, resulting in poor density of the composite at the edge. On the other hand, it may be due to a poor sintering process, resulting in the insufficient discharge of residual gas at the edge of the SD/Cu-Fe sandwich composite.

## 5. Conclusions

Aiming at the cutting tool used for cutting stone under complex working conditions such as high-load rough machining and intermittent cutting with large impact, the SD/Cu-Fe composite with the “sandwich” structure was designed, and its micro- and macro-characteristics were studied. The main conclusions included:

Within the sintering temperature range of 740–780 °C, diamond particles were relatively evenly distributed in the copper matrix and the crystal form of diamond particles was relatively complete. The interface between diamond particles and the copper matrix, and the interface between the copper matrix and iron interlayer were in good condition, and there was no obvious gap.

According to the experimental measurement results, with the increase in sintering temperature, the density, hardness, flexural strength, and wear amount of AD/Cu-Fe sandwich composites increased first and then decreased, and the properties reached the best when the sintering temperature was 770 °C. With the gradual increase in temperature, the interior of the SD/Cu-Fe composite became denser, the interface between diamond and the copper matrix was better bonded, and its mechanical properties were optimized. When the sintering temperature reached 780 °C, the excessive sintering phenomenon would cause interface defects between the diamond and the copper matrix, and the mechanical properties would decrease. The density, hardness, flexural strength, and wear amount were 7.114 g/cm^3^, 94.9%, 68.1 HRC, 1076 MPa, and 0.015 g, respectively. Diamond plays the role of fixing dislocations in the process of plastic deformation, improving the hardness and flexural strength of composite materials, hindering further grinding of abrasive particles, and reducing the damage of abrasive particles to the matrix material.

## Figures and Tables

**Table 1:** Macroscopic hardness of SD/Cu-Fe sandwich composite.

Sintering Temperature	740 °C	750 °C	760 °C	770 °C	780 °C
The Macrohardness (HV)	640	650	900	940	700

**Table 2:** Flexural strength of SD/Cu-Fe sandwich composite.

Sintering Temperature (°C)	740	750	760	770	780
The Flexure Strength (MPa)	793.6	947.1	1047.0	1076.0	1066.0

## Data Availability

Not applicable.
